# Translation, cross-cultural adaptation, and validation of the Chinese version of self-efficacy and attitudes for providing Mouth Care scale

**DOI:** 10.1371/journal.pone.0271800

**Published:** 2022-07-22

**Authors:** Lan Chen, Liyan Gu, Xianchen Li, Wenyao Chen, Lingjuan Zhang

**Affiliations:** 1 Nursing Department, Shanghai General Hospital Affiliated to Shanghai Jiao Tong University School of Medicine, Shanghai, China; 2 Education and Scientific Research Department of Clinical Nursing, Changhai Hospital Affiliated to Naval Medical University, Shanghai, China; 3 Department of Neurology, NO. 905 Hospital of PLA Navy, Shanghai, China; 4 Clinical Research Center, Shanghai General Hospital Affiliated to Shanghai Jiao Tong University School of Medicine, Shanghai, China; 5 Shanghai Quality Control Center of Geriatric Care, Shanghai, China; International Medical University, MALAYSIA

## Abstract

**Background:**

In recent years, oral care for older people has received extensive attention in long-term care facilities. The Self-Efficacy for Providing Mouth Care (SE-PMC) and Attitudes for Providing Mouth Care (A-PMC) scale evaluated the self-efficacy and attitude of nursing staff while providing oral care. However, whether this scale is valid and reliable for Chinese nursing staff in China remains unverified. This study aims to translate the English version of SE-PMC and A-PMC into Chinese and determine their reliability and validity.

**Methods:**

After obtaining the author’s consent, the procedure for a double-back translation and cross-cultural adaptation was conducted to develop the Chinese version of SE-PMC and A-PMC. The validity and reliability of the Chinese version of SE-PMC and A-PMC were evaluated in a cross-sectional observational study with 852 nurses from 42 Geriatric Care Facilities (GCFs). Exploratory factor analysis (EFA) (n = 427) and confirmatory factor analysis (CFA) (n = 425) were conducted to test the construct validity and quality of the factor structures. We applied the item discrimination test and homogeneity test for item analysis. Cronbach’s alpha coefficient and split-half coefficient were adopted to evaluate internal consistency.

**Results:**

The Chinese version of SE-PMC (11 items, 3 factors) and A-PMC (11 items, 2 factors) included 22 items, reflecting adequate construct validity and reliability. In addition, test-retest reliability was 0.809 for SE-PMC and 0.811 for A-PMC, evincing good stability. The Cronbach’s α coefficient of SE-PMC was 0.831, with each factor ranging from 0.793~0.906. The Cronbach’s α coefficient of the A-PMC was 0.768, with each factor ranging from 0.814~0.824. Item-Content Validity Index (I-CVI) of SE-PMC and A-PMC ranged from 0.84 ~1.00 and 0.82~1.00, respectively.

**Conclusion:**

The Chinese version of SE-PMC and A-PMC was validated as a reliable assessment tool to evaluate the self-efficacy and attitude of nursing staff in GCFs for providing oral care in China.

## Introduction

As an essential part of the quality of life, oral health is closely related to the overall well-being of the older population [1, 2]. Because of the impact of dementia, disability, comorbidity, and palliative care, the oral health of older people in geriatric care institutions is facing both visible and invisible challenges [3]. In institutionalized populations, problems such as missing teeth, caries, tooth pain, periodontitis, oral infection, and dysphagia are common [3–5]. The clinical oral assessment study showed that institutionalized residents’ oral hygiene was poor since about 80% had plaques on the surfaces of their teeth [6]. A study in Germany reported that 48% of nursing home residents were edentulous, and 52% were at risk for malnutrition with dementia as a strong predictor [7]. According to the fourth national survey in China, 71.6% of 4332 older persons had caries, while 64.5% had periodontal pockets and 47.6% had unrestored tooth spaces [8]. Oral disease burden was reported to be associated with poor cognitive and physical functioning in the FINORAL study, a cross-sectional observational study investigating 209 residents’ oral status, functioning, and nutrition in long-term care facilities in Helsinki [9]. Persistent routine dental attendance and permanent tooth loss were detected as predictors of improvement and worsening oral health-related quality of life among older people in Sweden [10].

According to Sheeran, self-efficacy and attitude influence behaviour, cognition, and emotional processes and affect the coping strategies of healthcare staff [11]. The practical application of oral care in aged care facilities does not fully comply with established nursing guidelines and practices. For example, a study in Canadian nursing homes found that 59% of the care providers reported short time to provide oral care during the night shift, while 19% failed to complete oral care in time as scheduled [12]. As determined by Kistler, the primary perceived obstacles to oral care were residents’ reluctance to care and lack of time [13]. In another study, Wretman [14] believed oral care self-efficacy and the attitude of nursing staff are closely related to oral care quality. Therefore, it is necessary to evaluate nurses’ self-efficacy and attitude in providing oral care to provide informative data for oral healthcare research.

In order to investigate beliefs about oral care tasks among nursing staff in home-dwelling older people, the Dental Coping Beliefs Scale (DCBS) was developed thirty years ago [15] and then modified as the Nursing Dental Coping Beliefs index in Sweden [16]. The validity of the nursing DCBS index has been tested among staff in nursing homes [17, 18] but still seems to fail to meet the bar of conceptual and psychometric rigor. Aro [19] recently developed an instrument to measure nurses’ self-efficacy beliefs, challenges, and knowledge regarding oral health care in home care settings on a small sample showing relatively low validity. Chinese scholars have also tried to measure geriatric self‐efficacy for oral health [20], but the development of instruments for nurses’ oral care competence needs more scientific rigor [19]. Fortunately, the Self-Efficacy for Providing Mouth Care (SE-PMC) and Attitudes for Providing Mouth Care (A-PMC) scales developed by Wretman [14] have been applied in geriatric nursing institutions and verified to be reliable and valid.

At present, we did not find similar assessment tools in China. Therefore, this study aims to introduce, translate and validate the SE-PMC and A-PMC to evaluate the self-efficacy and attitude of nursing staff in providing oral care in geriatric care facilities (GCF) in China.

## Methods

### Study design and participants

We used cluster sampling and conducted a cross-sectional, descriptive survey of 42 GCFs in Shanghai, China. Totally 900 participants were recruited. According to the principle that the ratio of a sample size to items assessed is 1:10~1:20, a sample size of 220~420 participants is appropriate. In this study, we conducted both Exploratory Factor Analysis (EFA) and Confirmatory Factor Analysis (CFA), so the sample size was doubled. The inclusion criteria for participants were: (a) officially registered nurses in GCFs; (b) nursing experience in GCFs over one year; (c) informed consent and voluntary participation in this study. However, nurses who did not work in GCFs during the survey were excluded (off-site training or sick leave). The selection criteria for experts were: (a) experience over five years in nursing administration, clinical nursing, geriatric nursing, nursing education, or stomatology; (b) bachelor’s degree or above and senior professional title; (c) overseas educational background. This study was approved by the Institutional Review Board of General Hospital affiliated with Shanghai Jiaotong University, and written informed consent was signed by participants

### Instruments

#### Demographic characteristic

A self-designed form about the participants’ sociodemographic information included gender, age, length of work experience, and the characteristics (name, ownership, size) of the GCF.

#### The SE-PMC and A-PMC scales

The SE-PMC and A-PMC scales were developed by Wretman [14] in 2020. The researchers designed a questionnaire of 35 original items and surveyed 434 nurses in 14 nursing homes in North Carolina. After two years of follow-up, the SE-PMC and A-PMC scales were revised and formed to measure the self-efficacy and attitude toward providing oral care. The SE-PMC (11 items; Guttman’s λ2 coefficient 0.78) has three identified factors: ’ Promoting Oral Hygiene’, ’Providing Mouth Care’, and ’Obtaining Cooperation’. The A-PMC (11 items; Guttman’s λ2 coefficient 0.77) has two factors: ’ Care of Residents’ Teeth’ and ’Care of Own Teeth’. The scale is a self-rating scale with a first-person perspective. Options are rated on a 4-point Likert scale ranging from "1-strongly disagree" to "4-strongly agree" with a maximum score of 88 points and a minimum of 22 points. The higher the score, the better the self-efficacy in providing oral care. Participants could fill out the scales within 10 minutes.

#### Translation, adaptation, and psychometric testing

By email, we contacted the original author, Dr Wretman, and obtained permission to translate. Then, we translated and tested the SE-PMC and A-PMC based on the cross-cultural adaptation guidelines of the American Academy of Orthopedic Surgeons Evidence-Based Medicine Committee, including the five steps as follows.

*Forward translation*. The original scale was independently translated by two native bilingual researchers. One was a Ph.D. in nursing and had one-year visiting scholar experience in the United States. The other was a professional English teacher without medical background in a college.

*Synthesis*. After discussion and confirmation among the research group, a third native bilingual translator compared the two translated versions and formed the initial Chinese version of SE-PMC and A-PMC.

*Back translation*. The initial version was back-translated into English by two other researchers blinded to the original scale. Then, we compared the two back-translations and obtained a final Chinese translation.

*Evaluation of content validity*. We consulted 11 experts to integrate and culturally adjust the Chinese Version of SE-PMC and A-PMC.

*Pre-experiment*. The revised Chinese Version of SE-PMC and A-PMC was pilot tested in 20 nurses in a GCF selected by convenience sampling. We assessed whether the items could be easily understood and filled out. Then, the psychometric properties of the translated scales were estimated using item analysis, constructive validity and model fit, internal consistency reliability, and split-half reliability. Questionnaires (n = 852) were collected and randomly divided into the EFA group (n = 427) and CFA group (n = 425) automatically by SPSS 23.0 software.

### Data collection

From February to March 2021, 900 registered nurses from 42 GCFs in Shanghai were recruited. Informed consent was obtained before the investigation. We issued an online survey to collect data via Wen Juanxing (www.wjx.cn). A total of 900 questionnaires were recovered in the study anonymously. Due to the considerate settings of the online survey system, there were no missing items from the submitted 900 questionnaires, but 48 of them were invalid (option selection all "1" or all "4"). Therefore, 852 questionnaires were valid, and the effective recovery rate was 94.67%.

### Statistical analysis

SPSS 23.0 IBM and Mplus7.4 were used for data analysis. The mean ± standard deviation and median/quartile were used for normally and non-normally distributed continuous variables. Categorical variables were described using frequency and percentage. T-test was used for comparison between continuous variables. P<0.05 was considered statistically significant. Item discrimination test and homogeneity test were applied for item analysis. The internal consistency and homogeneity of the Chinese version of SE-PMC and A-PMC were assessed using Cronbach’s alpha (considering values over 0.70 as appropriate). An expert panel evaluated the content validity. Constructive validity was analyzed by EFA [20, 21], using principal component analysis with varimax rotation. CFA was performed to evaluate the validity further, adopting Root Mean Square Error of Approximation (RMSEA), the ratio of chi-square to degrees of freedom (χ^2^/df), Tucker-Lewis Index (TLI), Comparative Fit Index (CFI), and standardized root-mean-square residual (SRMR). For this study, the following criteria were used to evaluate model fit: χ 2 /df < 3.0, CFI >0.95, RMSEA < 0.06 and SRMR < 0.08, which suggest a good fit. Chi-square χ 2 /df < 5.0, CFI > 0.90, RMSEA < 0.08 and SRMR <0.10 suggest an adequate fit [22]. The reliability analysis adopts the internal consistency analysis (Cronbach’s alpha coefficient and Guttman split-half coefficient) [11, 12].

## Results

### Sample characteristics

Among the 852 participants, the average age was 30.27 years (SD, 7.24), and the majority were female (99.1%). The proportion of nurses working in public and private institutions is 50.7% and 49.3%, respectively. The average length of working experience was 5.91 years (SD, 5.93). Detailed sample characteristics are shown in Table 1.

**Table 1:** Characteristics of sample and settings (n = 852).

variables	EFA Group (n = 427)	CFA Group (n = 425)	Total Sample (n = 852)
	n (%) or mean ± SD	n (%) or mean ± SD	n (%) or mean ± SD
Age (years)	30.41±7.17	29.99±7.63	30.27±7.24
Gender			
male	5 (1.2)	3 (0.7)	8 (0.9)
female	422 (98.8)	422 (99.3)	844 (99.1)
Type of institution			
Public	225 (52.7%)	207 (48.7%)	432 (50.7%)
Private	202 (47.3%)	218 (51.3%)	420 (49.3)
Experience of Work	6.08±5.79	5.7±6.09	5.91±5.92

EFA = Exploratory factor analysis; CFA = confirmatory factor analysis; SD = standard deviation

### Item analysis

We ranked the total score of submitted questionnaires and compared the high-scored group (the top 27%) and low-scored group (the last 27%) of the Chinese version of SE-PMC and A-PMC separately, using an independent-sample t-test. For the Chinese version of SE-PMC and A-PMC, the CR value was 10.39~13.48 and 4.76~14.84. The correlation coefficient between each item and the total score ranged from 0.531 to 0.657 in SE-PMC and 0.316 to 0.696 in A-PMC.

### Content validity

In this study, experts used a 4-point Likert scale to evaluate the relevance of each item, from 1 being "not relevant" to 4 being "very relevant". The results showed that the Chinese version of SE-PMC and A-PMC had the I-CVI ranging from 0.82 to 1.00 and the S-CVI/UA of 0.89, indicating good content validity.

### Constructive validity and model fit

EFA was used for constructive validity to determine whether the scale was suitable for factor analysis. The KMO (Kaiser-Meyer-Olkin) test (value = 0.832) and Bartlett’s sphericity test (χ2 = 2469.528, df = 55, P<0.0001) of the Chinese version of SE-PMC showed common factors exist and are suitable for factor analysis. Principal component analysis and varimax rotation were used to extract factors. As a result, three factors were identified in SE-PMC with eigenvalues above 1.0 (4.577, 2.002, and 1.458), accounting for 73.065% of the variance with factor loadings varying from 0.708 to 0.885. In the Chinese version of SE-PMC, factor 1, factor 2, and factor 3 comprised items S1~S5, S6~S8, and S9~S11, respectively. Moreover, the KMO value of the Chinese version of A-PMC was 0.817 with significant Bartlett’s sphericity test results (χ2 = 1890.642, df = 55, P<0.0001). Two factors were identified in A-PMC (eigenvalue 3.269 and 3.108), accounting for 57.966% of the variance, with factor loadings varying from 0.502~0.879. In the Chinese version of A-PMC, factor 1 and factor 2 comprised items A1~A6, and A7~A11, respectively. The factor loading matrices of the Chinese versions of SE-PMC and A-PMC are shown in Table 2.

**Table 2:** Factor loading matrix of the Chinese version of SE-PMC and A-PMC (n = 427).

Items	Factor 1	Factor 2	Factor 3
S1	**.818**	.257	.091
S2	**.783**	.249	.047
S3	**.854**	.108	.081
S4	**.863**	.142	.039
S5	**.851**	.159	.077
S6	.059	.001	**.836**
S7	.095	.097	**.880**
S8	.054	.086	**.801**
S9	.201	**.860**	.045
S10	.137	**.885**	.061
S11	.319	**.708**	.099
A1	**.730**	.080	
A2	**.800**	.021	
A3	**.502**	.203	
A4	**.879**	.086	
A5	**.767**	.072	
A6	**.688**	.152	
A7	.028	**.833**	
A8	.084	**.862**	
A9	.013	**.669**	
A10	.046	**.759**	
A11	.013	**.752**	

**Fig 1:**
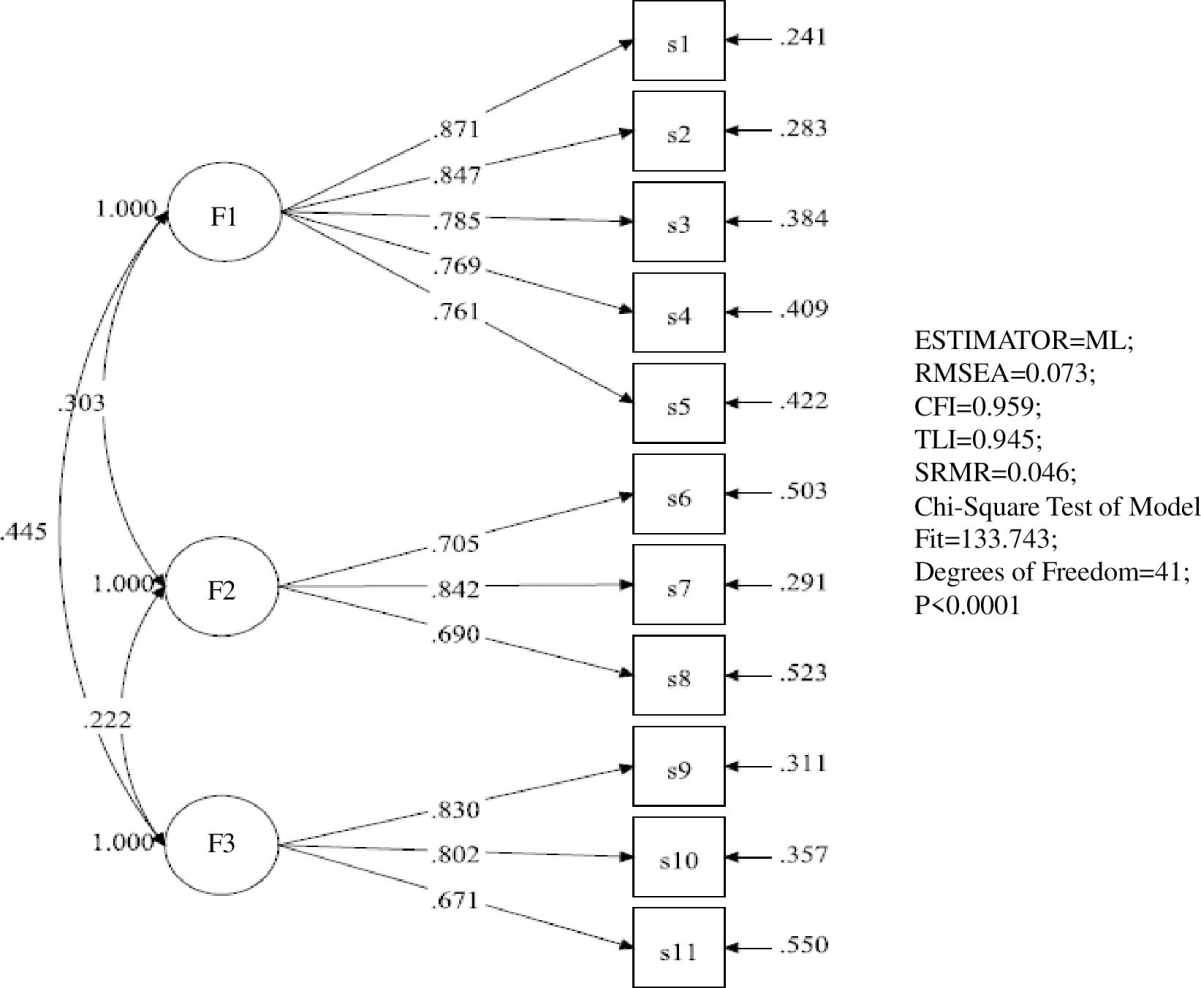
Confirmatory factor analysis of the Chinese version of SE-PMC. F1: Promoting Oral Hygiene; F2: Providing Mouth care; F3: Obtaining Cooperation.

**Fig 2:**
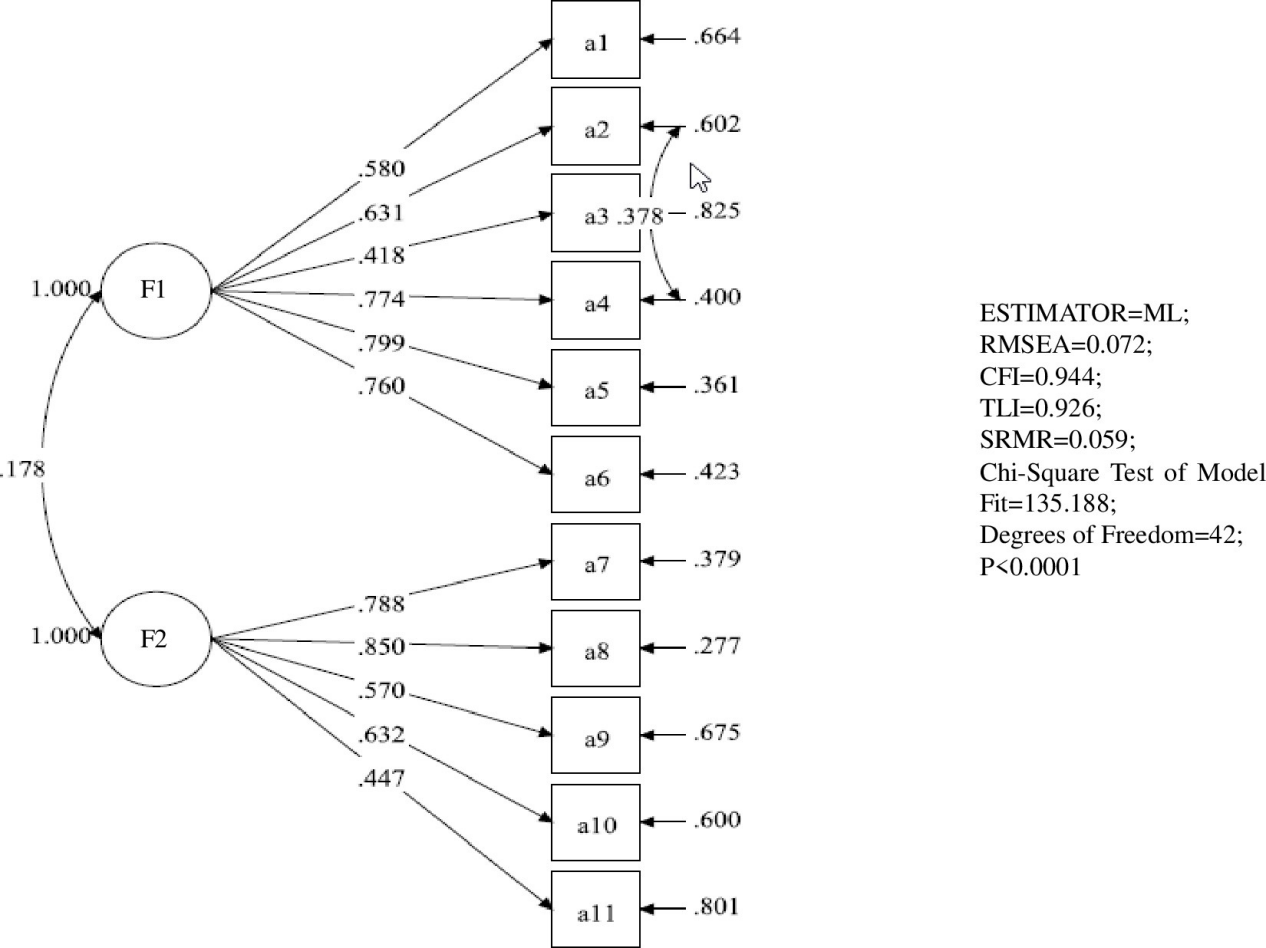
Confirmatory factor analysis of the Chinese version of A-PMC. F1: Care of Residents’ Teeth; F2: Care of Own Teeth.

Then, we conducted CFA to verify the three-factor and two-factor model using another sample of 425 participants. The items of the Chinese version of SE-PMC were estimated by the maximum likelihood method, and Promoting Oral Hygiene (POH), Providing Mouth care (PMC), and Obtaining Cooperation (OC) were used as latent variables to draw a path diagram to form a SE-PMC model, as shown in Fig 1. For the entries of the Chinese version of A-PMC, Care of Residents’ Teeth (CRT) and Care of Own Teeth (COT) were used as latent variables to draw a path map to form an A-PMC model and then modified (see Fig 2). The fit indices of the initial and the modified model are shown in Table 3. The CFA results demonstrated that the two models met the requirement of a standardized estimate.

**Table 3:** Model fit indices from the confirmatory factor analysis (n = 425).

Model	χ^2^	df	SOME	TFI	CFI	RMSEA (90% CI)	P
SE-PMC	133.743	41	0.046	0.945	0.959	0.073 (0.059–0.087)	<0.001
A-PMC	170.534	43	0.061	0.901	0.923	0.084 (0.071–0.097)	<0.001
A-PMC modified	135.188	42	0.059	0.926	0.944	0.072 (0.059–0.086)	<0.001

### Internal consistency and split-half reliability

For the Chinese version of SE-PMC and A-PMC, Cronbach’s alpha coefficient was 0.831 and 0.768, while Cronbach’s alpha coefficient of sub-dimensions ranged from 0.793~0.906 and 0.814~0.824, respectively. The respective Guttman split-half coefficients of the two scales were 0.809 and 0.811. The reliability results of the scales and each dimension are shown in Table 4.

**Table 4:** Reliability analysis results of the Chinese version of SE-PMC and A-PMC (n = 852).

	Total (n = 852) Cronbach’s α	EFA Group (n = 427) Cronbach’s α	CFA Group (n = 425) Cronbach’s α
SE-PMC	0.831	0.832	0.829
POH	0.906	0.909	0.904
PMC	0.793	0.798	0.788
OC	0.811	0.813	0.808
A-PMC	0.768	0.772	0.764
CRT	0.824	0.822	0.825
COT	0.814	0.835	0.790

## Discussion

This study aimed to assess the validity and reliability of the Chinese version of SE-PMC and A-PMC. The translation and application of the tool could help evaluate caregivers’ self-efficacy and attitude in GCFs in providing oral care. Cross-cultural adaptation was carried out under the AAOS-recommended guidelines. In addition, the selection criteria for translators and experts were strict. Furthermore, participants in this study came from 42 GCFs in Shanghai, representative of both public and private institutions, which further supported our results’ reliability.

Item discrimination test and homogeneity revealed the items applicable in the Chinese version scales. The CFA results indicated that the translated tool possesses stable structures. The test-retest reliability showed stability across time. Therefore, the Chinese version of SE-PMC and A-PM has good reliability and validity results, consistent with those of the original English version.

Four items were modified as follows during the translation and adaptation of SE-PMC and A-PMC.

*Item S4*: "If I brush and floss residents’ teeth correctly, I expect they will experience fewer dental problems." was modified as "If I clean and floss correctly for the older people, they will experience fewer dental problems."
*Item S5*: "I believe I can help independent residents have better oral care." was revised to "I believe I can provide better oral care to the older people who can take care of themselves."
*Item S10*: "I know ways to successfully provide oral care to residents who hit or scream." was adjusted to "I know how to successfully provide oral care to screaming, aggressive seniors."
*Item A3*: "If residents’ gums bleed, I feel I should probably stop brushing their teeth altogether." was modified as "If the older person has bleeding gums, I should probably stop brushing altogether."

Self-efficacy influences behaviour, cognition, and emotional processes [23, 24], while attitudes are related to job performance and quality of care [25, 26]. As poor oral hygiene is a marker for poor health-related quality of life, long-term care facilities need oral care education from caregivers and regular dental check-ups [6]. However, caregivers often face various challenges due to ineffective communication, the uncertainty of risks, and comorbidities of older people, causing pressure and powerlessness to provide oral care [9]. Therefore, it is necessary to evaluate the self-efficacy and attitudes of Chinese caregivers in GCFs since they are often exposed to long-term work, time constraints, weak awareness, and insufficient training [27].

In a previous study, researchers admitted that caregivers in communities consistently played an important role by enhancing oral health knowledge, maintaining positive attitudes, increasing older persons’ ability to perform oral self-care, and enhancing oral self-care awareness [28]. Currently, there are assessment scales focusing on older persons’ or periodontal patients’ oral health status in China, usually using oral health self-efficacy as a sensitive indicator [29, 30]. Academic research targeting the oral health of Chinese older persons was mostly studies exploring relations between oral health and general conditions, malnutrition, quality of life, cognition, and sarcopenia. [31–33]

Moreover, caregivers’ theoretical education and clinical skill refinement had the highest potential to enhance long-term outcomes, as reported by Chicote [34]. Likewise, Wretman [14] proposed a positive relationship between SE-PMC and A-PMC scores and residents’ oral hygiene. In their study, not-for-profit nursing homes and staff with fewer years of experience reported higher scores. Similarly, our study also showed differences among staff and institutions, indicating that the Chinese version measurements were helpful in oral hygiene quality improvement and promotion programs.

As noted, the translation and adaptation of SE-PMC and A-PMC into Chinese has potential clinical implications for oral healthcare research in Chinese populations. The current research would appear to fill the gap between the practical quality of oral care and the need for oral hygiene promotion from caregivers’ perspectives in GCFs. Further application in larger samples is desired to validate the utility of the tools better.

## Limitations

Although our results support the translated tool’s reliability and validity, several limitations still exist. Firstly, the sample of the nursing staff was recruited from institutions in Shanghai, China. Those institutions are equipped with more human resources and advanced devices; thus, the findings may not represent all caregivers in GCFs in China. Secondly, since the SE-PMC and A-PMC are self-reported, social desirability bias appears unavoidable in the responses. Although participants in our study fulfilled the questionnaires online and anonymously, some might choose the options according to administrators’ expectations. Thirdly, most of the participants were female who might have higher sense of professional identity than male staff, which could lead to the bias of results. Future research is needed to extend the translated instrument’s application further to verify its reliability, validity, and stability.

## Conclusions

As revealed by our results, the Chinese version of SE-PMC and A-PMC has good reliability and validity. It can be used to measure the self-efficacy and attitude of oral care provided by nursing staff in GCFs in China. The research team’s next step is to add qualitative interviews with nurses based on the quantitative results from the investigation to grasp a deeper understanding of the possible facilitators and barriers to providing oral care.

## Supporting information

S1 Checklist STROBE statement—checklist of items that should be included in reports of observational studies. (DOCX)

S1 Dataset (XLSX)

S1 File (PDF)

S2 File (PDF)
