# Supplementary material for: Translation, cross-cultural adaptation, and validation of the Chinese version of self-efficacy and attitudes for providing Mouth Care scale
Source: PLoS One. 2022 Jul 22;17(7):e0271800. doi: 10.1371/journal.pone.0271800 (PMC9307152; doi:10.1371/journal.pone.0271800)
Supplement: S1 File — (PDF) [file pone.0271800.s003.pdf]

上海市第一人民医院医学伦理委员会科研项目伦理批件

批件编号　2020KY040	审查日期　2020.7.10		项目编号　2020 科 040
项目名称	老年护理机构口腔健康促进方案干预效果的研究		
项目来源	自筹		
主要研究者	陈兰		
研究单位	上海市第一人民医院	科室	护理部

审查文件（含版本号）如下：

（1）　研究方案

（2）　知情同意书

（3）　主要研究者履历

1、审查方式

☐会议审查　　　■快速审查　　　☐紧急会议审查

2、审查结果

同意

3、该研究进行过程中将接受伦理委员会的持续审查？■是　　☐否

审查频率为该研究批准之日起每 12 月一次。

伦理委员会有根据实际进展情况改变持续审查频率的权利。

4、批件有限期为 36 个月，至 2023 年 7 月 10 日 止。

医院伦理委员会（盖章）：

日期：2020 年 7 月 10 日

上海市第一人民医院人体试验伦理审查委员会

地址：上海市海宁路 100 号（200080）　　　电话：（021）63240090

扫描全能王 创建
