# Supplementary material for: Translation, cross-cultural adaptation, and validation of the Chinese version of self-efficacy and attitudes for providing Mouth Care scale
Source: PLoS One. 2022 Jul 22;17(7):e0271800. doi: 10.1371/journal.pone.0271800 (PMC9307152; doi:10.1371/journal.pone.0271800)
Supplement: S2 File — (PDF) [file pone.0271800.s004.pdf]

Ethical Approval for Scientific Research Projects from the Medical Ethics Committee of Shanghai First People's Hospital

上海市第一人民医院医学伦理委员会科研项目伦理批件

批件编号 2020KY040<br>Approval No.	审查日期 2020.7.10<br>Date of review		项目编号 2020科040<br>Project No.
项目名称	老年护理机构口腔健康促进方案干预效果的研究<br>Research on the effect of interventions in oral health promotion programmes in elderly care facilities		
项目来源	自筹 Source of project: Self-financing		
主要研究者	陈兰 Principal Investigator: Chen Lan		
研究单位	上海市第一人民医院	科室	护理部

审查文件（含版本号）如下： Research Institution: Shanghai First People's Hospital, Department of Nursing

（1） 研究方案

（2） 知情同意书

（3） 主要研究者履历

The following documents (with version numbers) were reviewed:
(1) Study protocol
(2) Informed consent form
(3) Principal investigator's curriculum vitae

1、审查方式 Mode of review

meeting review √rapid review emergency meeting review

☐会议审查 ■快速审查 ☐紧急会议审查

2、审查结果 Review results

Consent

同意

3、该研究进行过程中将接受伦理委员会的持续审查？■是 ☐否

审查频率为该研究批准之日起每 12 月一次。

伦理委员会有根据实际进展情况改变持续审查频率的权利。

Will the study be subject to continuous review by the ethics committee during the course of the study? √Yes No
The frequency of review is once every 12 months from the date of approval of the study.
The Ethics Committee has the right to change the frequency of ongoing review according to the actual progress.

4、批件有限期为 36 个月，至 2023 年 7 月 10 日 止。

The approval has a limited duration of 36 months until 10 July 2023.

医院伦理委员会（盖章）：

日期：2020 年 7 月 10 日

上海市第一人民医院 人体试验伦理审查委员会

地址：上海市海宁路 100 号（200080） 电话：（021）63240090

扫描全能王 创建
